# Drug-induced liver injury prediction based on graph convolutional networks and toxicogenomics

**DOI:** 10.1371/journal.pcbi.1013423

**Published:** 2025-09-05

**Authors:** Tong Xiao, Ying Liu, Kaimiao Hu, Kaimin Guo, Mengying Zhang, TingTing Wang, Weihua Lei, Wenjia Wang, Shuiping Zhou, Yunhui Hu, Ran Su

**Affiliations:** 1 School of Computer Software, College of Intelligence and Computing, Tianjin University, Tianjin, China; 2 Tianjin Tasly Digital Intelligence Chinese Medicine Technology Co., Ltd., Tianjin, China; 3 State Key Laboratory of Chinese Medicine Modernization, Tianjin, China; University of Rochester Medical Center, UNITED STATES OF AMERICA

## Abstract

Drug-induced liver injury is a leading cause of high attrition rates for both candidate drugs and marketed medications. Previous in silico models may not effectively utilize biological drug property information and often lack robust model validation. In this study, we developed a graph convolutional network embedded with a biological graph learning (BioGL) module—named BioGL-GCN(Biological Graph Learning-Graph Convolutional Network)—for drug-induced liver injury prediction using toxicogenomic profiles. The BioGL module learned the optimal graph representations of gene interactions by utilizing the constructed protein-protein interaction network, which represents initial gene relationships, and gene frequency information obtained from gene enrichment analysis. Finally, the graph convolutional network was used to identify drug hepatotoxicity. Our method pays more attention to gene-gene relationships compared to previous approaches, thereby achieving more accurate predictive performance. We applied BioGL-GCN to predict DILI risk for active components in the integrated traditional Chinese medicine (ITCM) database and validated these predictions through hepatotoxicity experiments using a 3D primary human hepatocyte (PHH) model. The results showed that our model achieved a prediction accuracy of 79%, thus further validating the reliability of the constructed model.

## Author summary

Drug-induced liver injury is a major challenge in drug development, often leading to costly late-stage failures or even drug withdrawal. In this study, we developed a new computational model called BioGL-GCN to predict whether drugs are likely to cause liver damage. Our approach combines graph-based machine learning with toxicogenomic data—information that reflects how genes respond to drug exposure. Unlike previous methods, BioGL-GCN takes into account the interactions between genes, which improves its prediction accuracy. To further validate our approach, we tested BioGL-GCN on natural compounds from traditional Chinese medicine, which often have complex structures that are difficult to evaluate using standard methods. Using a 3D human liver cell model, we confirmed that our predictions matched real-world toxicity outcomes with precision 79%, significantly higher than conventional approaches. By bridging computational modeling and experimental validation, our research provides a practical tool for early-stage drug safety assessment. It has the potential to reduce both the risks and costs associated with the introduction of new medicines to the market.

## Introduction

Drug-induced liver injury (DILI) often leads to rejections of new drug applications, forces pharmaceutical companies to adjust dosing guidelines and issue medication warnings, and sometimes results in the withdrawal of drugs from the market [1,2]. Between 1990 and 2010, 133 drugs meeting the inclusion/exclusion criteria were withdrawn from the market due to safety concerns, and 36 of these drugs (27.1%) were specifically recalled due to hepatotoxicity problems [3]. Considering the highly time-consuming nature and enormous costs associated with the development of new drugs [4], establishing an efficient and accurate predictive model for hepatotoxicity at the early stages of drug development is of significant importance.

Traditionally, the hepatotoxic properties of xenobiotics are determined using a variety of “in vivo” and “in vitro” models. However, these models are time-consuming, expensive, and operationally complex. In contrast, “in silico” approaches have garnered significant interest among researchers for predicting human DILI risk given their cost-effectiveness and ease of implementation. Therefore, many in silico quantitative structure-activity relationship (QSAR) models have been developed to predict DILI risk [5–8]. In recent years, deep learning-based methods originally designed for drug-target interaction (DTI) or affinity prediction have also inspired new directions in computational toxicology [9,10]. While these methods demonstrate promising performance, they primarily focus on molecular and protein sequences rather than transcriptomic responses. Furthermore, despite these advances, most existing in silico models face two challenges: (1) limited sample sizes in training datasets [11,12], and (2) the absence of standardized DILI classification criteria for consistent annotation.

The development of robust and accurate in silico DILI prediction models is critically dependent on a large data set of drugs with reliable DILI classifications. High-throughput screen technologies have enabled the generation of toxicogenomic profiles for millions of compounds at an incredibly lower cost by monitoring hundreds to thousands of genes simultaneously. The National Institutes of Health (NIH) developed the LINCS L1000 dataset [13], which captures over 1.3 million toxicogenomic profiles using high-throughput screening technologies to measure gene expression levels. This large amount of data could improve the robustness and accuracy of the DILI model. The US Food and Drug Administration (FDA) developed an annotation scheme to label the DILI risk of 1,036 FDA-approved drugs, announcing the DILIrank [14] dataset in 2016. DILIrank categorizes drugs into four classes: most-DILI concern, less-DILI concern, no-DILI concern, and ambiguous DILI concern. It stands as the predominantly employed resource for developing DILI prediction models and has been widely incorporated in various scholarly investigations [15–17]. In 2020, the FDA further enhanced DILIrank to create DILIst [18] (Severity and Toxicity of Liver Injury) by incorporating four additional literature datasets. Until now, DILIst is the largest dataset with DILI classification, containing 1,279 drugs and providing an invaluable resource for predicting DILI risk. Ting Li et al. developed an eight-layer Deep Neural Network (DNN) model for DILI prediction using the LINCS L1000 dataset with DILIst [19]. Despite significant progress in the prediction of DILI, existing computational approaches still face two key challenges. First, biological networks, as typical graph-structured data in non-Euclidean space, present complex topological structures that make conventional deep learning models, such as DNN, difficult to apply directly. Second, there remains a critical need for effective strategies to integrate multi-source biological knowledge and automatically learn optimal graph representations that can support advanced feature extraction and predictive analysis using graph convolutional networks (GCNs).

GCNs have emerged as a powerful architecture for learning node (or graph) representations [20]. Within the field of bioinformatics, numerous variants of GCNs have emerged and garnered particular attention [21–25]. Nevertheless, the application of GCNs is often constrained by the type of input graph, primarily originating from two categories: fixed biological networks, such as known protein-protein interaction (PPI) networks, which are clearly defined within specific biological domains, or artificially constructed graphs such as those created through Gaussian kernel k-nearest neighbor graphs [26]. Since many of these graphs rely on domain knowledge or human design, evaluating whether they are optimally suited for the semi-supervised learning efficiency of GCNs presents a challenge. This is because these graph structures may not adequately align with the core requirements of GCNs [27]. Su et al. proposed a graph convolutional network equipped with a graph learning (GL) module, termed glmGCN, for predicting distant metastasis in cancer [28]. They used a PPI network to represent the initial relationships between genes and then employed the GL module to learn the optimal graph representation of gene interactions. Compared to previous GCN-based approaches, this method pays closer attention to gene-gene relationships, thereby achieving more accurate predictive performance.

Building upon these advancements, we proposed BioGL-GCN, a graph convolutional network embedded with a bioinformatics graph learning (BioGL) module. In BioGL-GCN, we modeled toxicogenomic profiles as graph-structured data and employed the BioGL module to dynamically learn an optimized graph representation. Importantly, the BioGL module incorporated gene frequency information derived from enrichment analysis into the graph construction process, enabling the model to capture functionally relevant gene interactions more effectively. This method emphasized the incorporation of biologically relevant knowledge and the optimal understanding of gene relationships, thereby enhancing predictive performance. To further evaluate our model’s generalization, we applied it to predict hepatotoxicity of active components in the integrated traditional Chinese medicine database using their expression profiles and validated these predictions with a 3D primary human hepatocyte liver toxicity model. Our model achieved 79% accuracy, significantly outperforming the 42% accuracy of the SMILES-based method, demonstrating its competence in predicting hepatotoxicity of natural substances with complex molecular structures.

## Results

### Overview of the proposed approach

**Fig 1:**
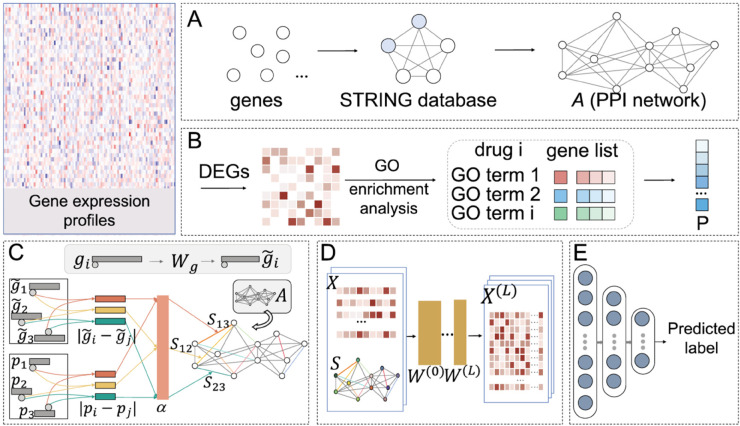
Overview of the proposed BioGL-GCN. (A) PPI network construction; (B) gene frequency extraction; (C) biological graph learning; (D) graph convolution; (E) output.

The overall framework of the proposed network is illustrated in Fig 1. The entire workflow comprises steps including (A) PPI network construction, (B) gene frequency extraction, (C) biological graph learning, (D) graph convolution and (E) output. Initially, we preprocessed the data by performing gene enrichment analysis and constructing the PPI network. Subsequently, we utilized the proposed architecture to acquire novel patterns of gene interactions and extract features. Finally, fully connected layers were used to map the distributed feature representations into the label space to complete prediction of DILI risk.

### Visualization of the features

**Fig 2:**
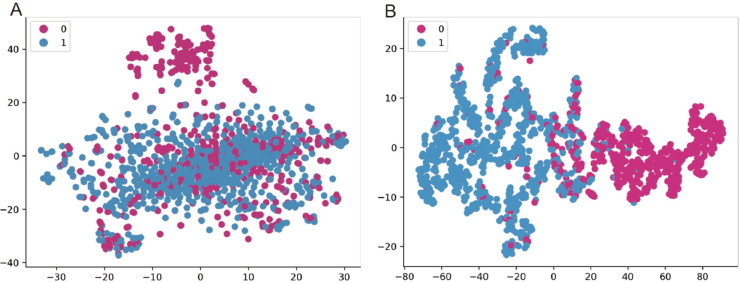
T-SNE visualization based on raw features and features extracted from BioGL-GCN; 0 represents the samples without hepatotoxicity and 1 represents the samples with hepatotoxicity. We chose one fold to show the plots. (A) The results based on the raw features. (B) The results based on the features extracted from BioGL-GCN.

To visualize the features employed for prediction, we utilized the T-Distributed Stochastic Neighbor Embedding (T-SNE) method, a powerful dimensionality reduction technique that maps high-dimensional data into a low-dimensional space while preserving the local structure of the dataset. Our primary objective was to assess whether the features extracted from the last fully connected layer of BioGL-GCN exhibit superior separability compared to the raw features. The experimental results are depicted in Fig 2. As shown in the figure, the distributions of the two labels are heavily overlapping and poorly separable when based on the raw features. In stark contrast, the features derived after the graph convolution operation exhibit a clear and distinct separation between the two labels, indicating that BioGL-GCN extracts discriminative features capable of enhancing prediction performance.

### Comparison of BioGL-GCN with other models

#### Compared with “non-deep” methods.

**Fig 3:**
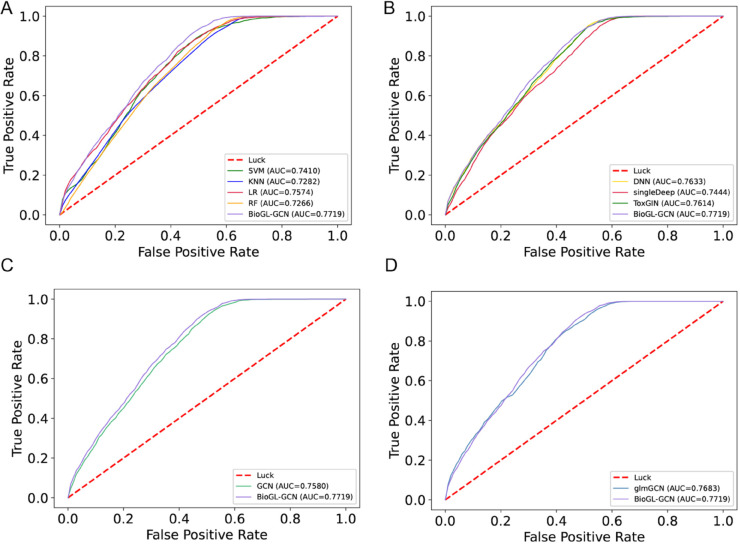
ROC curves of BioGL-GCN and other models: (A) Compared with “non-deep” methods; (B) Compared with deep methods; (C) Compared with GCN; (D) Compared with glmGCN.

We compared our approach with four “non-deep” machine learning methods, including Support Vector Machine (SVM), K-Nearest Neighbors (kNN), Logistic Regression (LR), and Random Forest (RF), which are highly popular in biomedical applications. Upon examining Table 1 and Fig 3A, we observed that among the four conventional machine learning methods, SVM performed the best, boasting a 74.01% accuracy rate and an AUC value of 0.7410. Our method has a slight improvement with the accuracy rate increased by 1.66% and the AUC value increased by 0.0309 over SVM. Moreover, our model exhibited a more balanced specificity and sensitivity. This indicates that our approach is more accurate in predicting DILI compared to conventional machine learning methods.

**Table 1:** Compared with other models.

Methods	Accuracy(%)	Specificity(%)	Sensitivity(%)	F1-score(%)	AUC
SVM	74.01	54.16	87.62	80.08	0.7410
KNN	72.52	42.72	92.82	80.07	0.7282
LR	70.75	60.11	78.00	76.02	0.7574
RF	72.02	48.85	87.81	78.87	0.7266
DNN	74.63	52.05	90.03	80.86	0.7633
GCN	73.38	55.43	85.60	79.28	0.7580
glmGCN	74.55	50.53	90.89	80.95	0.7683
singleDeep	75.25	42.01	97.87	82.47	0.7444
ToxGIN	75.33	51.76	91.37	81.51	0.7614
**BioGL-GCN**	**75.67**	**52.96**	**91.12**	**81.64**	**0.7719**

#### Compared with deep methods.

We also compared our proposed method with several state-of-the-art deep learning approaches, including DNN, singleDeep [29], and ToxGIN [30]. Unlike these methods that typically take raw gene expression profiles as input or rely on simplified network structures, our proposed BioGL-GCN constructed a gene-gene interaction graph, considering the aggregated effects of neighboring genes. As shown in Table 1 and Fig 3B, our proposed BioGL-GCN method significantly improved performance. Specifically, compared to DNN, we increased ACC by 1.04%, SPEC by 0.91%, SEN by 1.09%, F1 score by 0.78%, and AUC by 0.0086. Compared to singleDeep, BioGL-GCN improved ACC by 0.42% and AUC by 0.0275. In comparison with ToxGIN, our method increased ACC by 0.34% and AUC by 0.0105. Therefore, based on our experimental results, we concluded that incorporating graph-structured data containing gene-gene interaction information was more effective in improving prediction accuracy than relying solely on raw gene expression profiles.

#### Compared with GCN.

To evaluate the effectiveness of the BioGL layer, we compared our model against a standard GCN. Similar to BioGL-GCN, we fed the PPI network and gene expression data into the GCN network model to extract features, followed by fully connected layers and a softmax function for feature mapping and prediction. The distinction between BioGL-GCN and GCN lies in the depiction of gene-gene interactions, which corresponds to node-node relationships in the gene network graph. While GCN directly utilized the PPI network to encode gene interactions, BioGL-GCN augmented the GCN framework with the BioGL layer. This yielded a new pattern of gene interactions that integrated PPI information with gene similarity, thereby better representing the interactions between genes. From Table 1 and Fig 3C, it was evident that compared to GCN, BioGL-GCN led to improvements in accuracy by 2.29%, sensitivity by 5.52%, F1 score by 2.36%, and AUC by 0.0036. These experimental outcomes suggest that the unique gene-gene interactions derived from the BioGL layer afford deeper insight, thus leading to superior overall performance.

#### The impact of the improved BioGL layer.

In pursuit of an optimal and adaptable graph representation tailored for graph convolutional layers, our work introduced a BioGL layer that innovated upon the glmGCN framework proposed by Su et al. By incorporating gene frequency information, which indicated the significance of each gene within biological pathways and processes, the BioGL layer specifically highlighted the importance of gene interactions. We adopted the glmGCN model for comparison. Similar to BioGL-GCN, we fed the PPI network and gene expression data into the glmGCN network model to learn gene interaction graphs and extract features, and utilized fully connected layers and softmax for feature mapping and prediction. The distinction is that the glmGCN failed to include gene frequency information in this instance. As evident from Table 1 and Fig 3D, our model achieved an accuracy increase of 1.12%, specificity enhancements of 2.43%, F1 score increments of 0.69%, and an AUC uplift of 0.008. These results showed that incorporating gene frequency into the patterns of gene interactions learned by the graph learning layer could lead to more accurate DILI predictions.

### BioGL-GCN model captured critical DILI-related pathways

**Fig 4:**
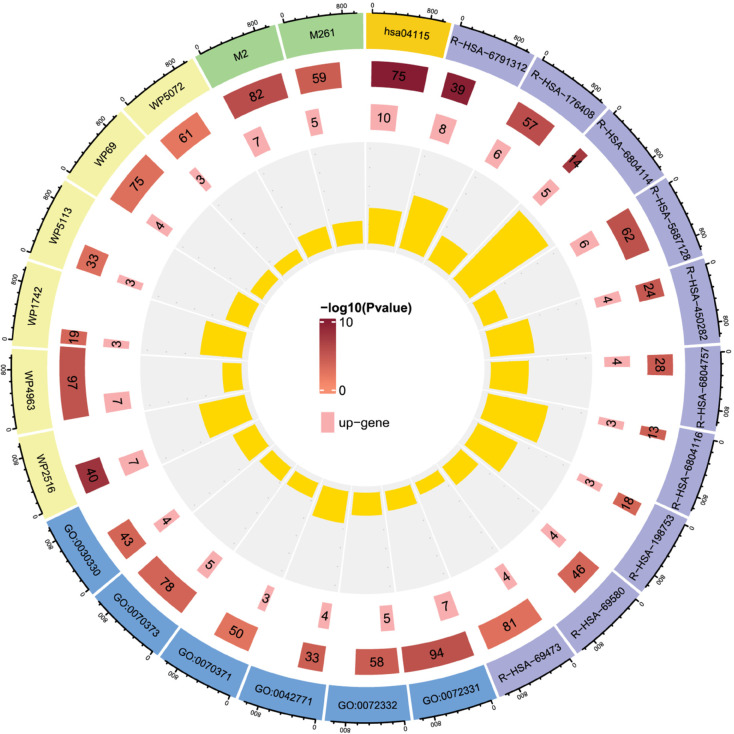
Significantly enriched pathways and processes for the top 200 high-frequency genes.

**Fig 5:**
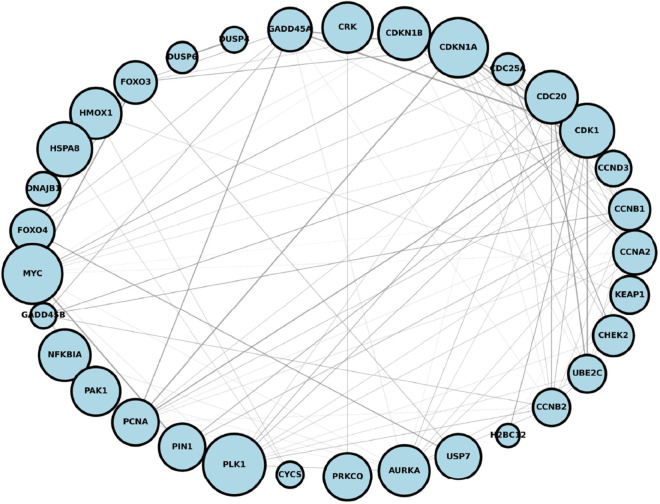
Distribution of DILI key pathway genes in the graph structure of the BioGL-GCN Model. The size of the node is projected based on the frequency of the gene. Edge thickness corresponds to the strength of gene–gene interactions.

We initially selected the best-performing fold of the predictive model and extracted the correctly predicted samples. From these, we further identified the top 200 genes with the highest frequency. Pathway and process enrichment analysis was performed using Metascape, which integrates multiple ontology sources (KEGG Pathway, GO Biological Processes, Reactome Gene Sets, Canonical Pathways, CORUM, WikiPathways, and PANTHER Pathway) to ensure comprehensive and reliable results. As shown in Fig 4, these high-frequency genes were significantly enriched in biological pathways related to DILI, particularly the p53 signaling pathway (KEGG Pathway: hsa04115). The p53 is a critical tumor suppressor that plays a pivotal role in regulating cell growth, DNA repair, and apoptosis. In the context of severe DNA damage induced by acetaminophen (APAP) overdose, p53 is activated to either inhibit cell proliferation or trigger programmed cell death. Furthermore, the p53 signaling pathway is closely associated with compensatory liver regeneration following APAP-induced acute liver injury [31]. Analysis of Reactome Gene Sets and GO Biological Processes revealed significant enrichment of multiple pathways related to cell cycle regulation, such as the regulation of APC/C activators between G1/S and early anaphase (Reactome Gene Sets: R-HSA-176408), as well as TP53-mediated G1 and G2 cell cycle arrest (Reactome Gene Sets: R-HSA-6804116 and R-HSA-6804114). These findings suggest that DILI may disrupt normal cell cycle progression, potentially leading to uncontrolled cell proliferation or cell death. Furthermore, significant enrichment was observed in the MAPK signaling pathway, particularly the MAPK6/MAPK4 signaling and ERK/MAPK targets (Reactome Gene Sets: R-HSA-5687128 and R-HSA-198753). This pathway dynamically regulates inflammatory responses, cell proliferation, and apoptosis, playing a dual role in liver injury repair: it can suppress excessive proliferation to prevent tumorigenesis, but may also exacerbate damage when dysregulated [32]. Finally, the G2/M DNA damage checkpoint (Reactome Gene Sets: R-HSA-69473), ATM signaling pathway (WikiPathways: WP2516), and PID SMAD2/3 nuclear signaling pathway (Canonical Pathways: M2) have also been identified as critical pathways in DILI [33,34]. Furthermore, to thoroughly investigate how these pathways influence prediction outcomes through the graph structure learned by BioGL, we visualized the distribution of genes from the identified key pathways within the learned graph structure *S*, as shown in Fig 5. The network visualization revealed that genes involved in critical pathways such as p53 signaling and MAPK signaling formed tightly interconnected subgraphs, with a global clustering coefficient of 0.54 and a graph density of 0.28, indicating a highly organized and functionally coherent architecture. These structural properties suggest that the model effectively captures biologically meaningful interactions among DILI-related genes, thereby enhancing its predictive capability.

### Experimental validation for active ingredients of Traditional Chinese Medicine (TCM)

**Fig 6:**
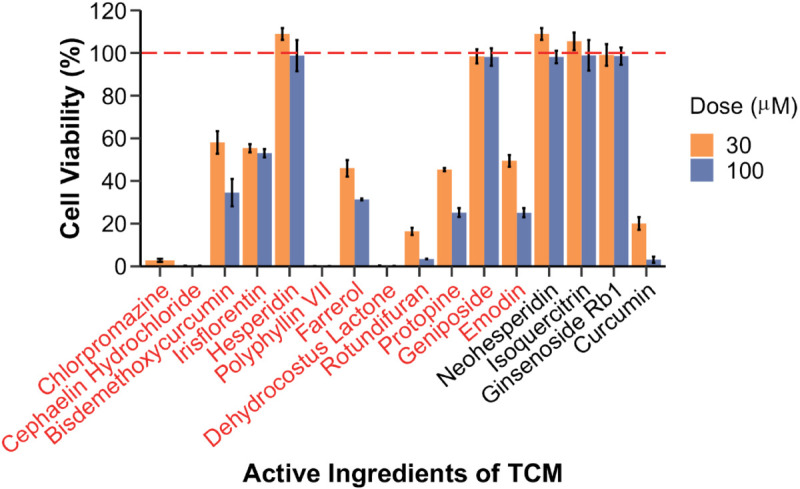
Validation of hepatotoxicity for 15 active ingredients of TCM based on the collagen-based 3D PHH model (Chlorpromazine used as a positive control in the 3D PHH model).

We obtained the probability of hepatotoxicity (DILI score) for 496 active ingredients of TCM based on the BioGL-GCN model (see S1 Table). The higher the probability of hepatotoxicity, the higher the DILI score. Following the sorting of the ingredients based on their DILI score in descending order, 11 ingredients with high probability (*DILIscore*>0.8) within the top 50 and 4 ingredients with low probability (*DILIscore*<0.4) were selected and validated using a collagen-based 3D PHH model [35]. The results showed that 9 out of 11 high-probability ingredients been confirmed to be hepatotoxic (accuracy: 0.82), while 3 out of 4 low-probability ingredients were validated as non-hepatotoxic (accuracy: 0.75) (Fig 6).

The overall prediction accuracy reached by 0.79. The results indicated that our model can effectively predict the hepatotoxicity of natural products with complex molecular structures based on their transcriptional profiles.

Additionally, we predicted the hepatotoxicity of the 14 ingredients using the online tool ADMETlab 3.0 (https://admetlab3.scbdd.com/) [36], which utilizes the SMILES representation of molecules as input. We classified a drug as hepatotoxic if its “DILI” or “Human Hepatotoxicity” score exceeded 0.5 by referring to classification provided by the website. The results indicated that 9 out of the 14 ingredients were predicted to be hepatotoxic, while 5 were predicted to be non-hepatotoxic. Among the 9 ingredients predicted to be hepatotoxic, only 5 were confirmed as such by the 3D PHH test, resulting in an accuracy of 0.55. Additionally, only 1 of the 5 ingredients predicted to be non-hepatotoxic was validated. This indicates that our model, based on transcriptional profiles, outperforms the SMILES-based method in predicting hepatotoxicity for ingredients with larger molecular weights and complex structures.

## Discussion

DILI is a critical safety consideration throughout the entire drug development process, encompassing all stages from preclinical to clinical studies [37]. Driven by the rapid development of high-throughput technologies like the L1000 and the establishment of standardized frameworks for DILI classification, such as DILIst, many machine learning and deep learning-driven studies have made substantial advancements. In spite of that, we notice that within the gene networks associated with drugs, there might exist latent biological attribute correlations among the genes themselves. In this study, we incorporated additional biological information (gene expression profiles, PPI and gene frequencies) to fully capture the relationships between genes, which met the core requirements of GCN and were then used for feature extraction and transformation. We compare BioGL-GCN with various approaches, encompassing GCN-based methodologies, deep methods, and four “non-deep” machine learning algorithms. The results demonstrate BioGL-GCN’s superior performance. We also observed that previous DILI prediction models often lacked robust and reliable experimental validation. To address this, we further validated our model using a 3D PHH model for liver toxicity experiments. The prediction results for active ingredients in TCM showed that our model exhibited high consistency with hepatotoxicity experiments using the 3D PHH model and outperformed SMILES-based methods. This not only validated the applicability of our model in predicting the hepatotoxicity of natural products with larger molecular weights and complex structures but also fully demonstrated the effectiveness and applicability of the graph learning layer we constructed based on biological expression information, as well as the methodological rigor of using GCN to predict DILI. Despite promising results, the small sample size (n=15, constrained by limited resources and experimental capacity) may limit the generalizability of our findings. Future studies will validate the model with a larger and more chemically diverse set of TCM ingredients.

## Materials and methods

### Data preparation

#### Toxicogenomic profiles for model development.

Ting Li et al. curated a drug-induced transcriptome profiles dataset from the NIH LINCS L1000 dataset. They matched the Level 5 transcriptomic data from LINCS L1000 with the DILIst database using the PubChem Identifier service based on drug names and synonyms, obtaining 23,791 transcription profiles involving 69 cell lines. An improved Kennard-Stone algorithm was then used to extract transcription profiles with maximum explanatory variance. We matched the dataset information table [19] provided by Ting Li et al. into the LINCS L1000 dataset, resulting in a total of 6,000 transcription profiles of 978 landmark genes from 640 drugs (of which 3,568 were DILI positive and 2,432 were DILI negative). In this context, each transcription profile signifies the treatment effect of a unique combination of drug, dosage, duration, and cell line.

#### Gene frequency extraction via gene enrichment analysis.

Su et al. proposed that the co-expressed genes in certain biological process (BP) for multiple drugs can be used as indicators of toxicity [38]. Furthermore, genes that played more significant roles in the enriched BP tend to appear more frequently in the BPs for the drugs. Based on this observation, we believed that gene frequency information could be integrated as an important feature into the construction of gene interaction graph. We integrated gene frequency information into the construction of the BioGL. This information was obtained through Gene Ontology (GO) enrichment analysis [39], enabling a better understanding of interactions within complex biological networks. Specifically, we employed rigorous bioinformatics methodologies to identify differential expression genes (DEGs) in cells under drug perturbation conditions. This involved conducting differential expression analysis on the gene expression profiles for each drug, where a gene was classified as a DEG if its normalized expression value had an absolute value meeting or exceeding a predefined threshold of 2. The threshold was directly adopted from the definition of signature strength provided by Subramanian et al. [13], where it is used to quantify the number of landmark genes showing significant expression changes. It has since been widely adopted in large-scale transcriptomic studies such as those using the L1000 platform. We calculated the BPs enriched in each drug transcription profile through the GO enrichment analysis. Specifically, we performed the analysis using the enrichGO function in the clusterProfiler R package, with both the p-value cutoff and q-value cutoff set to 1. This setting ensured that no significance filtering was applied, allowing us to retain all possible GO terms for subsequent gene frequency calculation. One gene might enrich more than once for all the BPs of one drug transcription profile. Assuming we had *M* drug transcription profiles and *N* genes, for drug transcription profile *j*, denoted by *D*_*j*_, the genes enriched in totally $\tau_{j}$ BPs. For the i-th gene, it enriched totally on $t_{i\_j}(t_{i\_j}\leq \tau_{j})$ BPs for *D*_*j*_.

$$p_{i}=\frac{\sum_{j=1}^{M}t_{i\_j}}{M},for\;i=1,2,\ldots ,N$$ (1)

Here, *p*_*i*_ represents the frequency of the i-th gene. The gene frequencies obtained for the 978 landmark genes were normalized in preparation for their use as inputs into the network.

#### Construct the PPI network.

It has been reported that PPI networks play a crucial role in cellular functions and biological processes. Graph theory has demonstrated significant effectiveness when applied to the study of PPI networks [40].

In this study, we utilized the PPI network to model gene relationships, where genetic interaction scores from the STRING database [41] were used to quantify the strength of connections between gene nodes. Specifically, we retrieved high-confidence gene interactions from STRING using a minimum confidence score of 0.7, which corresponded to the “high confidence” threshold recommended by the database. Based on these interactions, we constructed the adjacency matrix $A\in {\mathbb{R}}^{N\times N}$, where *N* was the number of genes (978 landmark genes from the LINCS L1000 dataset). If there was direct interaction between two genes, we recorded the score in matrix *A*;otherwise, we marked it as 0.

To preserve node self-features during graph convolution, we incorporated an identity matrix *I*, representing self-loop connections, into *A*, setting all diagonal elements to 1. The final PPI network derived from the LINCS L1000 dataset contained 4,818 high-confidence gene interactions among the 978 landmark genes.

#### Active ingredients of Traditional Chinese Medicine.

Compared to small molecules, plant-derived natural products typically feature larger and more complex molecular structures. To validate our module’s ability to effectively predict the hepatotoxicity of natural products based on drug transcriptional profiles, we collected FPKM-normalized gene expression data for 496 active ingredients of TCM from the ITCM platform (http://itcm.biotcm.net) [42]. The expression profiles were obtained via RNA-seq following treatment with each ingredient at a dose of 10 μM for 12 hours in MCF-7 cell line. For each ingredient, the expression profiles included 3 biological replicates. To ensure that the data was aligned with the training dataset (LINCS L1000 level 5 gene expression profiles) of model, we firstly employed the moderated z-score (MODZ) procedure used for LINCS L1000 data processing to derive a consensus replicate signature for each ingredient (including the blank control). Briefly, a pairwise Spearman correlation matrix was computed between the replicate signatures with trivial self-correlations being ignored (set to 0). Weights for each replicate were then computed as the sum of its correlations to the other replicates, normalized such that all weights sum to 1. Finally, the consensus signature was obtained by the linear combination of the replicate signatures with the coefficients set to the weights. This procedure could effectively mitigate the effects of uncorrelated or outlier replicates. Subsequently, to obtain the relative gene expression of each ingredient, we used the following formula:

$$Relative\;expression=\log_{2}\frac{CS_{ingredient}}{CS_{control}}$$ (2)

Here, *CS*_*ingredient*_ represents the consensus signature for ingredient and *CS*_*control*_ represents the consensus signature for control. The relative expression levels of the 978 landmark genes for each ingredient were extracted and input into the trained BioGL-GCN model to predict their hepatotoxicity.

### Architecture of the BioGL-GCN

#### Bio-graph learning layer.

The input to the GCN is denoted as $G\langle X_{gcn},A_{gcn}\rangle$, where *X*_*gcn*_ signifies the features of individual nodes and *A*_*gcn*_ represents the adjacency matrix that encodes the relational information between nodes. Su et al. proposed a novel approach, named glmGCN, which embeds a graph learning (GL) module within the GCN framework. This glmGCN integrated GL with GCN to attain enhanced graph representations and facilitated semi-supervised learning. First, the PPI network was employed to obtain the adjacency matrix *A*_*gcn*_, which represented the graphical relationships between genes. In the GL layer, given node information $X_{gcn}=\{x_{1},x_{2},x_{3},\ldots ,x_{n}\}\in {\mathbb{R}}^{n\times m}$, a non-negative function *S*_*glmGCN*_ was employed to represent the relationship between data points *x*_*i*_ and *x*_*j*_ based on their nodal distance connections. By combining the adjacency matrix *A*_*gcn*_ with the function *S*_*glmGCN*_, a new graph representation $G(X_{gcn},S_{glmGCN})$ was constructed. Inspired by Su et al., we also incorporated a BioGL module in our architecture to learn the optimal graph structure, as depicted in Fig 1C. In contrast to the approach of Su et al., our model was based on gene expression profiles and incorporated additional biological information—the frequency information of genes obtained through enrichment analysis—within the BioGL layer.

To optimally construct the neighborhood structure of the data, we devised a nonlinear function *S* based on the gene expression matrix $G\in {\mathbb{R}}^{M\times N}$, adjacency matrix $A\in {\mathbb{R}}^{N\times N}$, and gene frequency information $P\in {\mathbb{R}}^{N\times 1}$. We defined *g*_*i*_ and *p*_*i*_ as the gene expression level and gene frequency of the i-th gene, respectively. As mentioned, gene frequency information was regarded as an important feature of genes. Su et al. used $\left(g_{i}\;-\;g_{j}\right)$ to represent the distance between genes. Similarly, we considered that the same approach was applicable to gene frequencies. Since gene frequency is a one-dimensional measure, we used the product $\left(p_{i}\;-\;p_{j})\;\cdot \;(g_{i}\;-\;g_{j}\right)$ to represent the distance between the i-th gene and the j-th gene. Let $S_{ij}=c(g_{i},g_{j},p_{i},p_{j})$ represent the relationship between the i-th and j-th genes. Here, we reduced the dimensionality of *S* by performing calculations in a low-dimensional space parameterized by the projection matrix $W_{g}\in {\mathbb{R}}^{N\times d}$, where *d*<*N* , to enhance computational efficiency. The graph *S* was learned through the BioGL layer as follows:

$${\tilde{g}}_{i}=g_{i}W_{g},for\;i=1,2\ldots N$$ (3)

$$S_{ij}=c(g_{i},g_{j},p_{i},p_{j})=\frac{A_{ij}^{\phi }exp(\sigma (\alpha^{T}((p_{i}-p_{j})\cdot ({\tilde{g}}_{i}-{\tilde{g}}_{j}))))}{\sum_{j=1}^{N}A_{ij}^{\phi }exp(\sigma (\alpha^{T}((p_{i}-p_{j})\cdot ({\tilde{g}}_{i}-{\tilde{g}}_{j}))))}$$ (4)

Here, *A*_*ij*_ represents the connection between the i-th gene and the j-th gene in the adjacency matrix *A* derived from the constructed PPI network. *ϕ* is a tunable constant that, by raising *A* to the power of *ϕ*, emphasizes the importance of the initial graph and magnifies the distinction between strong and weak interactions. *σ* is an activation function, and *α* is a learnable parameter vector. Ultimately, a final softmax operation ensured that the learned graph *S* adhered to the following properties:

$$\sum_{j=1}^{N}S_{ij}=1,S_{ij}\geq 0$$ (5)

The weight vector *α* and *W*_*g*_ were optimized through the following loss function:

$$L_{1}=\sum_{i,j}^{p}\|(p_{i}-p_{j})\cdot ({\tilde{g}}_{i}-{\tilde{g}}_{j}){\|}_{2}^{2}S_{ij}+\gamma \|S{\|}_{F}^{2}+\beta \|S-A^{\phi }{\|}_{F}^{2}$$ (6)

Here, *γ* and *β* are two tunable constants, *F* denotes the Frobenius norm. If the distance $\|(p_{i}\;-\;p_{j})\;\cdot \;({\tilde{g}}_{i}\;-\;{\tilde{g}}_{j})\|$ between the i-th gene and the j-th gene is large, *S* should be relatively small. The second term serves as regularization to control the sparsity of the learned gene relationship matrix *S*, with *γ* being the regularization coefficient. We also incorporated the initial relationship matrix *A* of genes into the process.

#### Graph convolutional network.

Our proposed network, built upon GCN and incorporating the BioGL layer, utilized the BioGL layer to learn the graph representation *S*, which was subsequently used in the graph convolutional layers. The output of the graph convolutional layers can be calculated as follows:

$$X_{i}^{\left(l+1\right)}=\sigma (S\;\cdot \;X_{i}^{\left(l\right)}\cdot W^{\left(l\right)}),for\;i=1,2.\ldots n$$ (7)

Here, l=0,1...L−1;$X_{i}^{\left(l+1\right)}$is the output activation of the (l+1)-th layer. ${\text{W}}^{\left(\text{l}\right)}$ is a trainable weight matrix for each convolutional layer. $W^{\left(0\right)}\in {\mathbb{R}}^{1\times h(0)}$ is an input-to-hidden weight matrix for a hidden layer with *h*(0) feature maps. $W^{\left(L\right)}\in {\mathbb{R}}^{h(L-1)\times C}$ is a hidden-to-output weight matrix for a hidden layer with *h*(*L*–1) feature maps (*C* is the class number. Here, *C* = 2). And σ(·) denotes an activation function.

Assuming *y*_*i*_ denotes the true label of the i-th sample, and *z*_*i*_ indicates the probability predicted by the model that the i-th sample belongs to the positive class (designated as class 1). Cross entropy loss function was used here:

$$L_{2}=-\frac{1}{n}\sum_{i=1}^{n}[y_{i}log(z_{i})+(1-y_{i})log(1-z_{i})]$$ (8)

All parameters of the entire architecture were optimized through the following approach:

$$L_{BioGL-GCN}=L_{1}+\lambda L_{2}$$ (9)

The entire model encompassed an input layer, multiple hidden layers, and an output layer. The hidden layers consisted of a BioGL layer, two graph convolutional layers, and three fully connected layers. Batch normalization was employed after the BioGL layer and the two graph convolutional layers to optimize parameters, stabilizing the learning process. A Flatten layer was adopted to transform pooled features into a one-dimensional vector. Following this, the fully connected layers were used to map the distributed features, with softmax being employed for the final prediction.

The pseudo-code for the proposed method is outlined in Algorithm 1:

**Algorithm 1 Constructing the BioGL-GCN Model for Predicting DILI.**

**Input:**

Gene expression matrix *G*, hepatotoxicity labels *Y*

**Output:** The BioGL-GCN model for predicting DILI.

1: Construct the PPI network based on the 978 landmark genes,

obtaining the adjacency matrix *A*;

2: Include self-linkage relations: Form $A_{new}\leftarrow A\;+\;I$, where *I*

is the identity matrix;

3: Perform gene enrichment analysis to obtain the gene frequency

matrix *P*;

4: Split the data into training set Gtrain and testing set

Gtest;

5: Calculate the new pattern of gene interactions *S*;

6: Feed Gtrain, Ytrain, and *S* into the GCN and train the model;

7: Five-fold cross-validation;

### Validation of drug-induced hepatotoxicity based on the collagen-based 3D PHH model

We utilized a collagen-based 3D PHH model to validate the hepatotoxicity of the active ingredients of traditional Chinese medicine [35]. Briefly, an integrated biomimetic array chip (iBAC) for establishing a collagen-based 3D PHH model for the high-throughput prediction of DILI was designed and developed. The iBAC was geometrically designed in a commercialized 96-well format, with a three-layer structure, including a reservoir hole at the top, a 3D implanting hole in the middle, and and an ultra thin glass slide underneath. The 3D implanting hole was well designed for establishing the ECM-based models. A mixture of PHHs and collagen was seeded into the 3D implanting hole to construct a collagen-based 3D PHH model as a case. The standard reagents for 14 active ingredients of TCM were purchased from the National Institutes for Food and Drug Control. Firstly, we dissolved the reagent in dimethyl sulfoxide (DMSO) to prepare a 20 mM stock solution for storage. Before conducting the experiment, the stock of each reagent was diluted with cell culture medium to 30 μM and 100 μM, respectively. After cell activation, the reagent solutions at concentrations of 30 μM and 100 μM were added to the culture medium and incubated for 72 hours. At the end of incubation, the 3D cultured PHH were collected for cell viability assays. Biochemical assays assessment of cell viability was performed by determining the ATP content of PHH cultured in 3D model using 3D Cell Viability Assay according to the manufacturer’s instructions. Briefly, the CellTiter-Glo reagent and cell culture medium were mixed with a volume ratio of 1:1. Luminescence was detected on a multiplate reader (EnVision Multimode Plate Reader, PerkinElmer) and normalized to vehicle (DMSO) control.

## Supporting information

S1 Table The probability of hepatotoxicity (DILI score) for 496 active ingredients of TCM174 based on the BioGL-GCN model. (XLSX)
